# Disruptions in Hypothalamic–Pituitary–Gonadal Axis Development and Their IgG Modulation after Prenatal Systemic Inflammation in Male Rats

**DOI:** 10.3390/ijms24032726

**Published:** 2023-02-01

**Authors:** Vasilina Ignatiuk, Marina Izvolskaia, Viktoria Sharova, Liudmila Zakharova

**Affiliations:** Koltsov Institute of Developmental Biology, The Russian Academy of Sciences, Vavilov Street, 26, 119334 Moscow, Russia

**Keywords:** prenatal inflammation, HPG axis, GnRH neurons, critical periods of development, long-term effects, IL-6, IgG modulation

## Abstract

The development of the neuroendocrine system, including the hypothalamic–pituitary–gonadal (HPG) axis, is sensitive to environmental impacts during critical developmental periods. Maternal immune system activation by bacterial or viral infection may be one of the negative impacts. This study focused on the effect of systemic inflammation induced by lipopolysaccharides (LPS *E. coli*) on the HPG axis development in male rat offspring, corrected by the anti-inflammatory action of polyclonal IgG and monoclonal anti-interleukin (IL)-6 receptor antibodies (IL-6RmAbs). A single LPS exposure on the 12th embryonic day (ED) led to a decrease in the number of afferent synaptic inputs on gonadotropin-releasing, hormone-producing neurons in adult male offspring. LPS exposure on ED18 did not lead to such disruptions. Moreover, after the LPS injections on ED12, circulating follicle-stimulating hormone and sex steroid levels were reduced, and the gonadal structure was disrupted. A prenatal IL-6R blockade with IL-6RmAbs and polyclonal IgG reduced the negative effects of inflammation on fetal HPG axis development. Overall, the data obtained confirm the morphogenetic effect of inflammation on fetal HPG development and IL-6 involvement in these processes.

## 1. Introduction

The timely development of neurons synthesizing the gonadotropin-releasing hormone (GnRH), which is the key regulator of the hypothalamic–pituitary–gonadal axis, determines the reproductive abilities in mammals [1]. In the adult rat brain, the GnRH neurons are located in the septopreoptic area of the anterior hypothalamus, along the Diagonal Band of Broca, and in the organum vasculosum of the lamina terminalis areas, without forming clearly localized nuclei [2,3]. The fibers of the GnRH neurons project to the diencephalic median eminence, where they form axo-vasal synapses with the portal capillary bloodstream, connecting the hypothalamus with the pituitary gland [4].

The neural fibers secrete GnRH into the portal bloodstream with a certain frequency, obtaining the necessary GnRH concentration there for initiating pituitary gonadotropin secretion, which stimulates sex steroid secretion and gametogenesis in the testes and ovaries [2]. Many afferent synaptic inputs formed by neurons mediating various neurotransmitter brain systems are involved in GnRH secretion regulation in the pituitary of adult animals [5,6]. Kisspeptin [7], neurokinin B, and dynorphin [8] are synthetized by neurons of the arcuate nucleus connected with axo-somatic synapses and involved in the regulation of GnRH impulse secretion [5,9].

The bodies of neurons whose fibers form synapses directly on GnRH neurons are detected in diverse brain areas [2]. Their effects are mediated by such neurotransmitters as gamma-aminobutyric acid (GABA) [10], glutamate [11], monoamines [12], and melatonin [13,14]. It has been accurately suggested that they regulate the frequency of GnRH impulse secretion in the median eminence [2,3,15,16]. The number of synaptic inputs on GnRH neurons in females changes depending on the phases of the estrous cycle [9], while the number in males remains relatively constant [17].

Disruptions in HPG axis development may lead to sexual maturation problems and infertility. Systemic inflammation induced by infectious agents during the early stages of pregnancy can be one of the risk factors [6,18,19]. Maternal immune system activation by bacteria or their cell wall component, lipopolysaccharide (LPS), during early ontogenesis induces a cascade of proinflammatory cytokine synthesis and secretion, causing developmental impairment of the HPG axis in fetuses [6,20,21]. It was previously discovered that LPS (*E. coli*) injections in female mice during the early stages of pregnancy led to an increase in interleukin (IL)-6, leukemia inhibitory factor (LIF), and monocyte chemotactic protein-1 (MCP-1) levels in the mother and the fetus [21]. The increased levels of proinflammatory cytokines were accompanied by a suppression of GnRH neuron migration into the fetal brain. A delay in GnRH neuron migration into the forebrain may affect the establishment of these neurons’ afferent synaptic inputs, with consequent disruptions in crucial points of HPG axis development.

Since the consequences of the negative effects of inflammation on the developing HPG axis cannot always be traced, the therapy currently conducted in adults does not allow for the elimination of the detected fertility disorders. Various attempts are currently being made to prevent or suppress the negative effects of inflammation using the intravenous administration of immunoglobulins (IgM, IgG), both in animals and in humans [6,22,23,24].

The efficacy and safety of monoclonal antibody administration against various proinflammatory cytokines and their receptors in the treatment of autoimmune and chronic inflammatory diseases have been assessed in recent years [25]. Recombinant humanized monoclonal anti-IL-6 receptor antibodies (IL-6RmAbs) are used in the treatment of severe coronavirus infections, accompanied by high levels of proinflammatory cytokines [26]. However, the use of monoclonal antibodies during pregnancy requires special caution. Their approbation on experimental animals is necessary.

This study aims to investigate the effects of LPS-induced systemic inflammation and the anti-inflammatory effects of polyclonal IgG and IL-6RmAbs (IgG1) on the formation of afferent synaptic inputs of GnRH neurons, on gonadotropocyte functions, and on the gonadal development of adult rat male offspring during early ontogenesis.

## 2. Results

### 2.1. The Effect of Prenatal LPS Exposure on the Synaptic Input Number on the GnRH Neurons and in Adjacent Areas in the Septopreoptic Area in Adult Male Offspring

**Figure 1:**
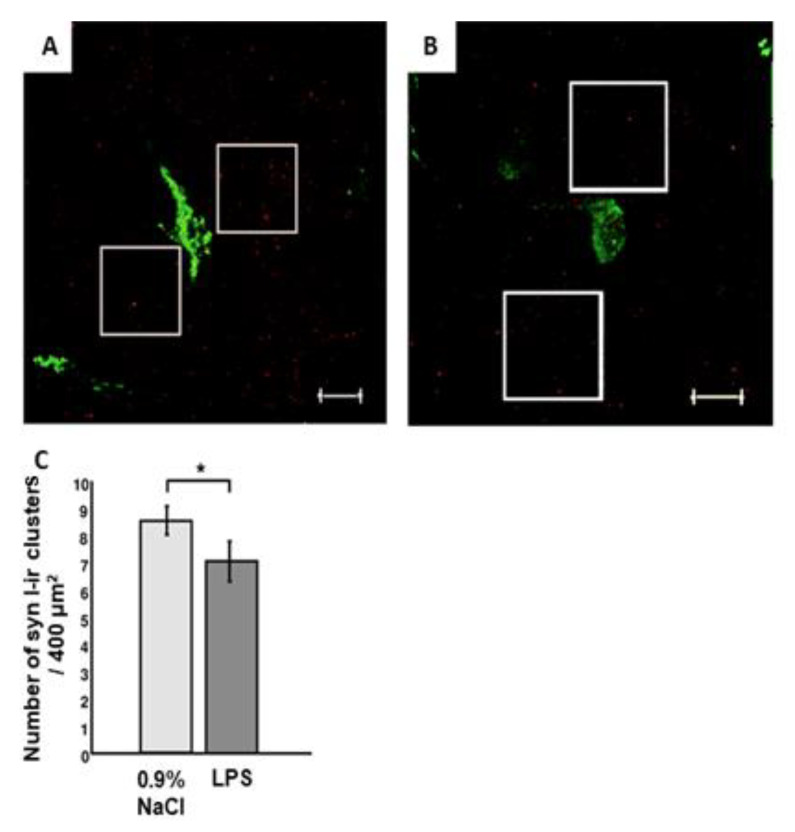
Synapsin-1-immunoreactive (syn 1-ir) clusters in areas adjacent to the GnRH neurons. Representative photomicrographs of male rat neurons on PND80 exposed on ED12 to (**A**) 0.9% NaCl (control) and (**B**) lipopolysaccharide (LPS, 50 μg/kg) stained with anti-GnRH (green) and anti-synapsin-1 (red) antibodies (n = 10 animals per group). Scale bar = 10 μm. (**C**) Average number of syn 1-ir clusters from two 400 μm^2^ areas (white box) placed at both sides of each neuron. M ± SEM, * *p* < 0.05 between groups. Approximately 10 visible GnRH-immunoreactive neurons on each of the 8–10 sections from each animal were analyzed.

Maternal LPS *(E. coli)* treatment on the 12th day of pregnancy (embryonic day 12, ED12) led to a significant decrease in the synapsin-1-immunoreactive cluster number in the areas surrounding the GnRH-immunoreactive neurons in the septopreoptic area of the anterior hypothalamus of male rats on postnatal day 80 (PND80) (Figure 1).

**Figure 2:**
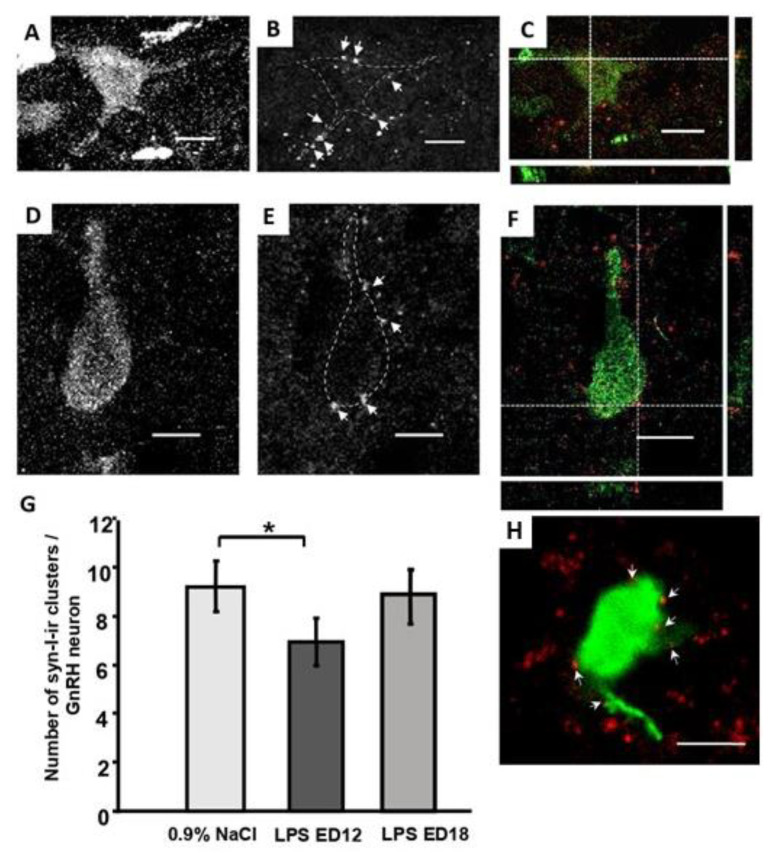
Synapsin-1-immunoreactive (syn 1-ir) clusters on the gonadotropin-releasing hormone (GnRH) neurons of males on PND80 exposed on ED12 to (**A**–**C**) 0.9% NaCl (control) and (**D**–**F**) lipopolysaccharide (LPS, 50 μg/kg). Maximum projection of confocal microphotograph stacks of neurons stained with anti-GnRH (**A**,**D**) and anti-synapsin-1 (**B**,**E**) antibodies. Neurons are outlined with dotted lines; white arrows mark the syn 1-ir clusters. Merge (**C**,**F**): orthogonal projections of single optical sections demonstrate the close apposition of syn 1-ir clusters with the GnRH neurons. Scale bar = 20 μm (**A**–**C**), 10 μm (**D**–**F**,**H**). (**G**) Number of syn 1-ir clusters in close apposition with the GnRH neurons of males exposed to saline or LPS on ED12 or ED18 (n = 10 animals per group). M ± SEM, * *p* < 0.05 compared with the control group. (**H**) Representative microphotograph of GnRH neuron of males treated with LPS on ED18.

GnRH-immunoreactive neurons were detected in the medial septal area of the forebrain, in the vascular organ of the lamina terminalis, and in the anterior hypothalamus of the diencephalon, both in LPS- and saline-exposed males. LPS exposure on ED12 led to a decrease (~30%) in the total number of synapsin-1-immunoreactive clusters on the bodies and dendrites of GnRH neurons in the studied areas of adult males, compared with the control group (Figure 2). Meanwhile, no disruptions in synaptic input formation were observed in males after LPS exposure on ED18 (Figure 2).

### 2.2. Long-Term Effects of IgG and IL-6 Receptor Blockade on Synaptic Input Formation on the GnRH Neurons of Male Rats after Prenatal Exposure to LPS

**Figure 3:**
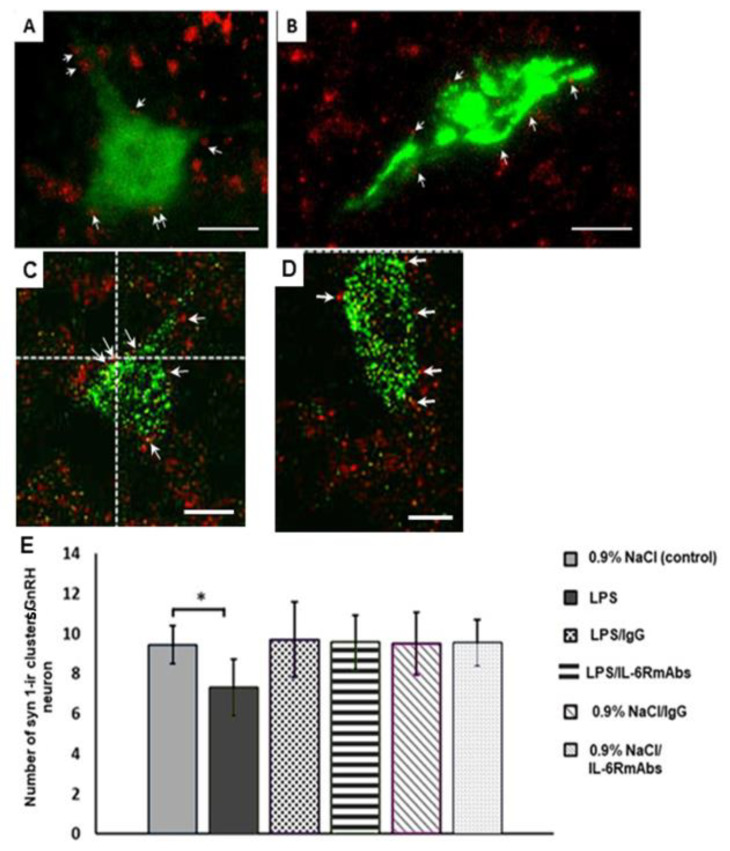
Synapsin-1-immunoreactive (syn 1-ir) clusters on the GnRH neurons of males on PND80 treated on ED12 with (**A**) LPS and IgG, (**B**) LPS and IL-6RmAbs, (**C**) 0.9% NaCl and IgG, and (**D**) 0.9% NaCl and IL-6RmAbs. The maximum projection of confocal microphotograph stacks of neurons stained with anti-GnRH (green) and anti-synapsin-1 (red) antibodies. The white arrows mark the syn 1-ir clusters. Scale bar = 10 μM. (**E**) Number of syn 1-ir clusters in close apposition with the GnRH neurons of males on PND80 treated on ED12 with 0.9% NaCl (control); LPS; LPS and IgG; LPS and IL-6RmAbs; 0.9% NaCl and IgG; and 0.9% NaCl and IL-6RmAbs (n = 10 animals per group). M ± SEM, * *p* < 0.05, compared with the control group.

Polyclonal IgG and IL-6RmAbs (IgG1) injected 40 min after LPS on ED12 led to a restoration of the synaptic input number on the GnRH neurons of adult male rats, compared to the control level (Figure 2 and Figure 3A,B,E). Administration of IgG and IL-6RmAbs to LPS-untreated pregnant females did not result in significant changes in the number of synapsin-1-immunoreactive clusters on the GnRH neuron bodies and dendrites (Figure 3C–E) of male offspring.

### 2.3. The Effect of Prenatal LPS and Immunoglobulin Treatments on the Plasma Levels of Follicle-Stimulating Hormone (FSH) and Sex Steroids in Adult Male Offspring

**Figure 4:**
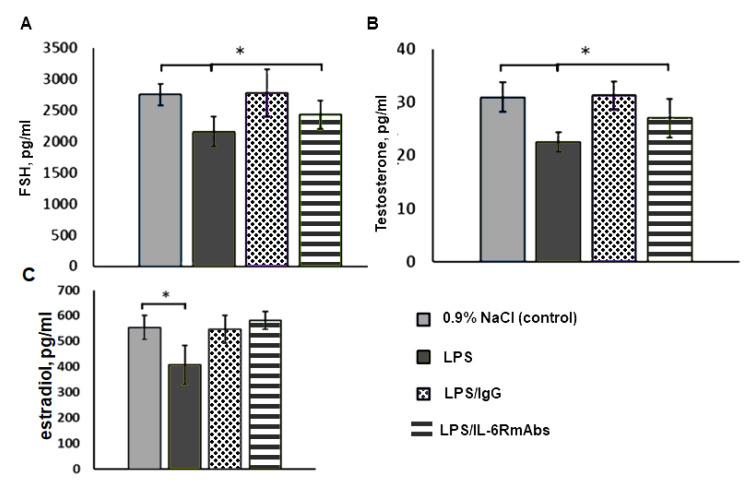
Plasma FSH (**A**), testosterone (**B**), and estradiol (**C**) levels in males on PND80 exposed on ED12 to 0.9% NaCl (control); LPS; LPS and IgG; and LPS and IL-6RmAbs (n = 10 animals per group). M ± SEM, * *p* < 0.05 between groups.

The maternal LPS exposure on ED12 led to a significant decrease in the FSH (Figure 4A), testosterone, and estradiol (Figure 4B,C) plasma levels of male rat offspring on PND80. Polyclonal IgG injection 40 min after LPS restored the levels of these hormones to the control levels. The IL-6RmAbs also restored the estradiol level to the control level (Figure 4C). The IL-6RmAbs injection led to a significant increase in the FSH and testosterone plasma levels of males, compared to the LPS-treated group. However, these levels remained significantly lower than in the control group (Figure 4A,B).

### 2.4. Long-Term Effects of LPS, IgG, and IL-6 Receptor Blockade on the Gonadal Structure in Male Offspring

**Figure 5:**
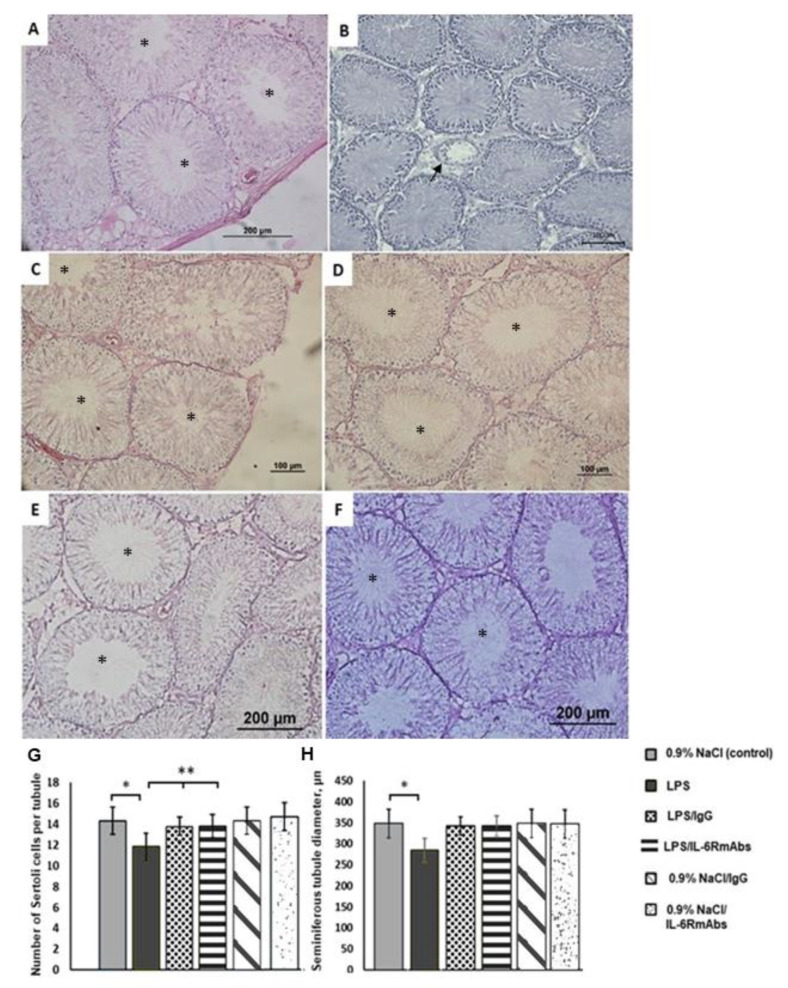
The morphology of testicular tissue on PND 80 exposed on ED12 to (**A**) 0.9% NaCl (control); (**B**) lipopolysaccharide (LPS, 50 μg/kg); (**C**) LPS and IgG; (**D**) LPS and IL-6RmAbs; (**E**) 0.9% NaCl and IgG; and (**F**) 0.9% NaCl and IL-6RmAbs. Hematoxylin/eosin staining. Scale bar = 100 μm. *—lumen, arrow—degrading tubule. (**G**) The number of Sertoli cells per seminiferous tubule (mean ± SEM) and (**H**) seminiferous tubule diameters in male rats on PND80 after exposure on ED12 to 0.9% NaCl (control); LPS; LPS and IgG; LPS and IL-6RmAbs; 0.9% NaCl and IgG; and 0.9% NaCl and IL-6RmAbs. M ± SEM. * *p* < 0.05, compared with the control group, ** *p* < 0.05, compared with the LPS-exposed group. A total of 50 randomly chosen round tubules from each animal were measured; Sertoli cells were counted in 30 round tubules from each animal; n = 10 animals per group.

The maternal LPS exposure on ED12 led to a significant decrease in seminiferous tubule diameter (F_3.88_ = 169.73, *p* < 0.01; n = 10 per group) and reduced the Sertoli cell number (F_3.88_ = 107.96; *p* < 0.01; n = 10 per group) in the testes of the male offspring on PND80, compared with the control group (Figure 5A,B,G,H). Most of the tubules did not have lumen; they were filled with cellular debris (Figure 5B). Such effects were not observed after LPS exposure on ED18.

Polyclonal IgG and IL-6RmAbs injected 40 min after LPS on ED12 led to the restoration of the seminiferous tubule diameters to the control level (Figure 5A,C,D,H). Moreover, a significant increase in the Sertoli cell number in the seminiferous tubules was observed (F_3.88_ = 95.49, *p* < 0.01, n = 10 males per group). However, their number remained lower than the control level (F_3.88_ = 12.58, *p* < 0.01; n = 10 males per group) (Figure 5A,G). The administration of IgG or IL-6RmAbs to LPS-untreated pregnant females did not result in significant changes in the number of Sertoli cells and seminiferous tubule diameters in male offspring (Figure 5E–H).

## 3. Discussion

The aim of the current study was to investigate the effects of systemic inflammation induced by LPS (*E. coli*) and the anti-inflammatory effects of IgG and IL-6RmAbs during the prenatal period on HPG axis establishment and function in male rat offspring. We focused on the remote effect of LPS and IgG on afferent synaptic input formation in males. According to our data, a single LPS administration on ED12 led to a decrease in the number of synaptic inputs on GnRH neurons (Figure 2). Meanwhile, LPS administration at later stages, corresponding to the intracerebral stages of GnRH neuron migration (ED18), did not lead to such disruptions. Reduced GnRH neuron innervation may be associated with disruptions in the development and functions of brain neurotransmitter systems, such as GABA, acetylcholine, and the melanin-concentrating hormone, which regulate the initiation of GnRH neuron intracerebral migration [27,28,29]. These signal molecules may act directly upon the migration of GnRH neurons to the forebrain by ED17, and the majority is located in this area, continuing their migration in this new environment. Various neurotransmitter system functions may be perturbed after exposure to different stressogenic factors, including bacterial and viral infections, which occur during early ontogenesis [18,30,31]. A decrease in the number of dopaminergic neurons in the substantia nigra, as well as in the quantity of serotoninergic neurons of myelencephalic raphe nuclei [32] and GABA-producing hippocampal neurons [33], was observed in adult rat and mouse offspring brains after prenatal LPS exposure during critical periods of brain development. This suggests that disruptions in the development of brain systems regulating GnRH neuron migration and functioning may affect the formation of GnRH neuron afferent innervation in male rats.

It should be noted that GnRH neuron functional activity regulation is mediated by an intricate neurohumoral system located not only in different brain areas but especially in the areas adjacent to GnRH neurons [10,34,35]. For instance, a reduced number of synaptic inputs both on GnRH neurons and in the adjacent areas resulted from prenatal dexamethasone treatment [36]. A reduced total synapse number in the forebrain, associated with an altered expression profile of synaptic proteins, was detected in rat offspring aged 2–3 months after various maternal stress impacts from ED13 to ED20 [37]. In this study, we detected a reduced number of synaptic inputs in areas adjacent to GnRH neurons in male rat offspring after maternal LPS exposure on ED12 (Figure 1).

The GnRH neurons are formed in the olfactory placode epithelium and migrate to the forebrain along the olfactory, terminal, and vomeronasal nerves on ED12-14 in rodents [29,38]. The process of migration can be divided into three stages: (1) intranasal migration, (2) penetration through the cribriform plate of the ethmoid bone, and (3) intracerebral migration. Each stage is characterized by a unique set of factors, such as cell adhesion proteins, gradients of guidance-cue molecules, and a specific cellular microenvironment producing neurotransmitters and neuromodulators [1,29]. Maternal LPS exposure on ED11,5-12, on the day of the beginning of GnRH neuron migration from the olfactory epithelium, led to a delay of their migration in mouse and rat fetuses [6,21,38]. The initial stages in the development of GnRH neurons are likely to be their critical period, since LPS exposure on ED15 did not induce any significant changes in the total neuron number or in their distribution between migration areas in rat fetuses [38]. On ED14,5-15 the GnRH neurons penetrated the cribriform plate [21]. On ED17-19 in rats, the majority of the GnRH neurons were located in the forebrain [38], and their development was regulated by factors different from the regulatory factors during the intramesenchymal stages (ED11,5-14). Notably, LPS exposure on neither ED15 [38] nor on ED18 induced such adverse effects as on ED12. This presents evidence that it was the initial stage of intranasal migration that was sensitive to LPS.

The disrupted migratory pathway formation and delayed migration of GnRH neurons, as well as the disrupted synaptogenesis in the brain, may be associated with elevated IL-6 synthesis induced by inflammatory processes during the prenatal period [21,39,40]. Under normal physiological conditions, IL-6 acts as a neurotrophic factor regulating neural differentiation and axonal growth [41]. Local inflammation induces elevated IL-6 synthesis, which may lead to developmental and functional disorders of various brain systems [32,42].

In summary, the IL-6R blockade with IL-6RmAbs during the prenatal period normalized the inflammation-induced abnormal changes in the number of synaptic inputs on GnRH neurons in adult male offspring (Figure 3).

The LPS-induced disruptions in the GnRH neuron synaptic input formation may impair the interplay between these neurons and the anterior pituitary gland and lead to the suppression of gonadotropocyte functions. After LPS exposure on ED12, reduced plasma FSH levels were detected in adult male rats (Figure 4A). It was previously demonstrated that luteinizing hormone (LH) levels did not change after prenatal LPS exposure in adult rats, although it was reduced during the juvenile period [43]. The pituitary FSH secretion was controlled by GnRH pulsatile secretion into the portal bloodstream. Notably, GnRH pulse frequency reduction stimulated FSH and suppressed LH secretion by the pituitary gonadotropocytes [44]. The above-mentioned disruptions in GnRH neuron afferent innervation may lead to GnRH pulse frequency changes and, consequently, to FSH level reduction.

Polyclonal IgG normalized the FSH plasma levels of adult males, which may be associated with the normalization of the number of synaptic inputs on GnRH neurons (Figure 4A). Meanwhile, IL-6RmAbs did not lead to a complete abolition of LPS negative effects, which indicates indirectly that other proinflammatory mediators are involved in this process.

The LPS exposure on ED12 also led to significant testis structure disorders (Figure 5). ED12 corresponded to the initial stages of Sertoli cell differentiation from proliferating somatic precursor cells, which interacted with migrating germ cells and regulated their subsequent pre- and postnatal development [45]. Cytokines, including IL-6 and LIF, are involved in the regulation of the Sertoli cell differentiation under normal physiological conditions [46]. An acute increase in proinflammatory cytokine levels associated with inflammation may disrupt the Sertoli cell differentiation.

Testis development continues postnatally, since during the juvenile period, a short-term HPG axis activation (“mini-puberty”) takes place [47]. It is associated with the replacement of testosterone-secreting fetal Leydig cells with an adult Leydig cell population [48]. During this period, testosterone and estradiol play crucial roles [49]. The LPS-induced testis disorders may be mediated by an elevated estradiol level in male rats during the juvenile period [18,43]. Meanwhile, in our study, adult offspring demonstrated suppressed testosterone and estradiol secretion (Figure 4B,C).

Regulation of spermatogenesis maintenance is the basic function of FSH in males. Sertoli cells, providing trophic, regulatory, and immunomodulatory factors for spermatogenesis [50], express FSH but not LH receptors [51]. Therefore, decreased FSH plasma levels may also account for structural disorders of the testes. The detected sex steroid level reduction in the male rat plasma could be explained by Leydig cell differentiation disorders, which are associated with a decrease in LH levels during the pre-pubertal and pubertal periods, as well as by GnRH pulsation frequency changes, which are associated with afferent innervation disruptions. Polyclonal IgG normalized the LPS-induced gonadal disorders (Figure 5C,D,G,H). Several mutually exclusive mechanisms of the immunomodulatory activity of IgG were previously proposed [52]. They include the binding of the IgG Fc fragment to the Fcγ receptors (FcγRs) expressed on innate immunity cells, which modulates their functions and FcγR expression; interference with the activation of the complement system or cytokine network cascade; the neutralization of autoantibodies; and the regulation of cell proliferation [53].

## 4. Materials and Methods

### 4.1. Animals and Experimental Design

This study was carried out on female Wistar rats initially weighing 200–260 g (Stolbovaya Breeding Center, Moscow, Russia) and their male offspring. Females were housed overnight with adult males from the same strain and supplier to obtain dated pregnancies. The day sperm was detected in a vaginal smear was considered ED1, while the day of birth was regarded as PND1. The animals were housed with 3–4 females per cage under specific pathogen-free standard conditions, with a 12 h light/dark cycle; they were supplied with ad libitum food and water. All manipulations of the animals were performed in accordance with the European Convention on the Protection of Vertebrate Animals Used for Experimental and Other Scientific Purposes (Strasburg 1986) and were approved by the Ethics Committee for Animal Research of the Koltzov Institute of Developmental Biology (the Russian Academy of Sciences, approval code: 23, approved on 15 November 2018).

**Figure 6:**
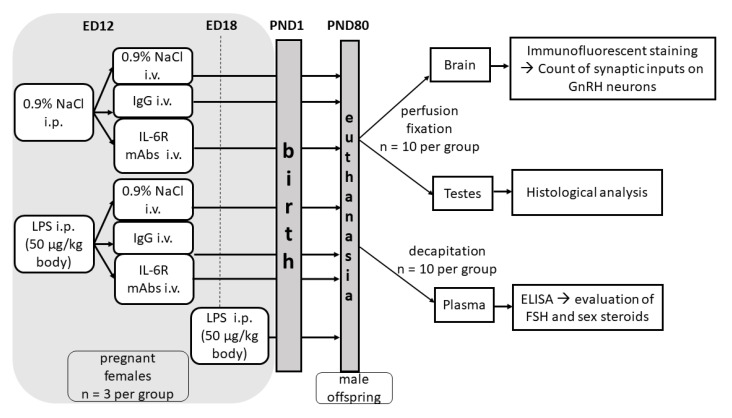
Scheme of the experimental design. A total of 18 pregnant rats (the first day of pregnancy was considered as ED1) were divided into two groups. On ED12, one group was injected intraperitoneally (i.p.) with 0.9% NaCl (control) and the second group with lipopolysaccharide (LPS; 50 µg/kg). At 40 min after the injections, the females of both groups were divided into 3 groups (n = 3 per group) and injected intravenously (i.v.) with 0.9% NaCl, IgG (1 mg/kg), or humanized IL-6RmAbs (IgG1; 2 mg/kg), respectively. A separate group of females (n = 3) was injected with LPS on ED18. On postnatal day 80 (day of birth was designated as PND1), male offspring born from all groups of females were transcardially perfused under anesthesia (n = 10 per group) to obtain brain and testes samples or decapitated (n = 10 per group) to collect plasma. Brain samples were stained with anti-GnRH and anti-synapsin-1 antibodies, and synaptic inputs on GnRH neurons were counted. Testes underwent histological analysis. Follicle stimulating hormone (FSH) and sex steroid concentrations were detected in blood plasma using ELISA.

On ED12, the females (n = 3 per group) were intraperitoneally (i.p.) injected with 500 µL of pyrogen-free 0.9% saline (control) or with LPS (*E. coli*, Sigma; 50 µg/kg body) in the same volume of saline. A separate group of females was injected with LPS on ED18. On ED12, 40 min after the LPS injection, when proinflammatory cytokine levels were not yet at their peak [21], the females were intravenously (i.v.) injected with 0.2 mL of 0.9% NaCl, rat IgG (Sigma-Aldrich, Saint-Louis, MO, USA, 1 mg/kg) in 0.2 mL of saline, or humanized IL-6RmAbs (IgG1) (2 mg/kg) (Chugai Pharma Manufacturing Co., Tokyo, Japan) in the same amount of saline. IgG and IL-6RmAbs were also injected into LPS-untreated animals (Figure 6). LPS and IgG concentrations were chosen according to previously published data [18,20,21,54]. Maternal LPS (50 μg/kg, i.p.) exposure did not lead to preterm labor or high embryonic toxicity, but it led to induced immune system activation, proinflammatory cytokine release, and fever. The IL-6RmAbs concentration was chosen experimentally. Pregnant females were injected i.v. with amounts of 8 mg/kg (recommended human therapeutic dose; n = 4), 4 mg/kg (n = 2), and 2 mg/kg (n = 3), and offspring survival rates were assessed. The concentrations higher than 2 mg/kg led to high embryonic mortality.

### 4.2. GnRH and Synapsin-1 Double Immunohistochemical Staining

Male rats (n = 10 per group) born from females of all the groups on PND80 were anesthetized by inhalation of Forane (Aesica Queenborough, Queenborough, UK). A transcardial perfusion was then performed with 0.01 M phosphate-buffered saline (PBS, pH 7.2–7.4) and 10 mL paraformaldehyde (4% PAF in 0.1 M phosphate buffer) sequentially. After decapitation, the brains were isolated and immersed in 4% PAF for 2 h at room temperature for postfixation. After that, the brains were immersed in a 25% sucrose (Sigma, St. Louis, MI, USA) solution in 0.02 M PBS for 36 h for cryoprotection and then frozen in hexane cooled to −40 °C with liquid nitrogen. The 10 μm thick frontal sections were prepared using a cryostat (Leica Microsystems, Wetzlar, Germany) at −23–25 °C, mounted on gelatin-coated glass slides, and dried at room temperature.

The sections were rinsed in 0.01 M PBS with 0.3% Triton X-100 (PBST; Sigma, USA) and incubated in a wet chamber sequentially with: (1) 1:15 fetal bovine serum for 1 h at +20 °C; (2) a mixture of primary rabbit polyclonal anti-synapsin-1 (AB1543, Abcam, Cambridge, UK, 1:500), which is a presynaptic marker [36] [Lim et al., 2016] and mouse monoclonal anti-GnRH I (sc-32292, Santa Cruz Biotechnology, Dallas, TX, USA, 1:500) antibodies in PBST containing 1% bovine serum albumin (BSA; Sigma, USA) for 48 h at +4 °C; and (3) a mixture of secondary antibodies—donkey anti-mouse IgG conjugated with AlexaFluor 488 (AB150105, Abcam, Great Britain; 1:500) and donkey anti-rabbit IgG conjugated with AlexaFluor 568 (AB175470, Abcam, Great Britain; 1:500) in PBST with 1% BSA (pH 7.2–7.4) for 2 h at +20 °C. The sections were rinsed three times for 10 min in PBST after the primary antibodies and in 0.01 M PBS after the secondary antibodies. After being rinsed, the samples were mounted in Mowiol (Sigma, CIIIA).

The sections were analyzed using a confocal microscope Leica TCS SP5 (Leica, Germany) using excitation wavelengths of 488 and 568 nm. Approximately 10 visible GnRH-immunoreactive neurons on each of the 8–10 sections from each animal were analyzed. Each neuron was scanned with a 60× immersion objective with a 4× digital zoom at 0.2 μm intervals, with constant laser intensity and detector sensitivity. Scans at each wavelength were performed sequentially across the optical sectioning to avoid bleed-through between the channels. The frame size of each GnRH neuron captured was set to 1024 × 1024 pixels, comprised of 50–80 stacks of serial images taken at 0.2 μm intervals through the entire depth of each neuron. The synaptic contacts were counted sequentially through the optical sections. The synapsin-1-immunoreactive clusters exhibiting close apposition with each GnRH-immunoreactive neuron body and dendrite were manually determined by scanning through the individual z-slices using Leica LAS AF (Leica, Germany) software. Synapsin-1-immunoreactive clusters were defined as being in contact with GnRH neurons if there were no pixels visible between the green and red fluorescence.

### 4.3. Gonadal Revising in the Male Offspring

After perfusion fixation, as described above, the testes were isolated and immersed in 4% PAF overnight at +4 °C. After that, the samples were incubated in 25% sucrose (Sigma, USA) solution in 0.02 M PBS for 36 h for cryoprotection and then frozen in hexane that was cooled to −40 °C with liquid nitrogen. Non-serial sagittal sections were prepared using a cryostat (Leica, Germany), mounted on gelatin-coated glass slides, dried, and stained with hematoxylin and eosin (H&E) using the standard procedures for morphological analyses. The sections were analyzed using a Keyence BZ-9000E microscope (Keyence, Japan) and the BZ Viewer (Keyence, Japan) software.

The randomly chosen, round-shaped, seminiferous tubules with a tubular diameter of 50 were measured on the sections from each animal (n = 10 per group). In each animal, the number of Sertoli cells per tubule was counted in 30 round-shaped tubules at the same stage of spermatogenesis (the elongated spermatid stage). The Sertoli cells were quantified on the basis of their previously reported morphological characteristics and their location in the tubules [54,55].

### 4.4. Evaluation of FSH and Sex Steroid Levels in the Male Offspring Blood by ELISA

On PND80, males (n = 10 per group) were decapitated, and their blood samples were collected in tubes with heparin and centrifuged (2000× *g*, 15 min, 20 °C) for plasma separation. Plasma FSH, testosterone, and estradiol concentrations were determined using Direct ELISA kits (Cloud-Clone Corporation, Katy TX, USA) according to the manufacturer’s instructions. The standard curves were plotted using the standards provided with the kits. The optical density was measured at 450 nm using a universal microplate reader (STAT FAX^®^ 2100 Awareness Technology, Inc., Palm City, FL, USA).

### 4.5. Statistical Analysis

The results were expressed as mean (M) ± SEM for each group. All data sets met the assumption of a normal distribution and homogeneity of variance. One-way analysis of variance (ANOVA) was used. A *p*-value of <0.05 was considered statistically significant. All analyses were performed using Statistica version 10 (Statsoft Inc., Tulsa, OK, USA).

## 5. Conclusions

The data obtained in this study suggest that LPS-induced systemic inflammation during the early stages of ontogenesis leads to HPG axis developmental disruptions, resulting in reproductive disorders in adulthood. After LPS exposure on ED12, which is likely to be a critical period of HPG axis development, the number of neural afferent inputs on GnRH neurons in the anterior hypothalamus and circulating FSH level were reduced, and testicular gameto- and steroidogenesis were suppressed in adult male offspring. The IL-6 receptor blockade with recombinant IL-6RmAbs diminished the negative effects of inflammation on HPG axis development, presenting evidence of IL-6 involvement in these processes. Forty min after LPS exposure, when the synthesis of proinflammatory cytokines, including IL-6, was not yet at its peak, polyclonal IgG, though non-specific, also modulated the inflammatory process.

## Abbreviations

BSA	bovine serum albumin
ED	day of embryonic development (embryonic day)
FSH	follicle-stimulating hormone
GABA	gamma-aminobutyric acid
GnRH	Gonadotropin-releasing hormone
HPG	Hypothalamic–pituitary–gonadal
IgG	immunoglobulin G
IL	interleukin
IL-6RmAbs	monoclonal anti-interleukin-6 receptor antibodies
LH	luteinizing hormone
LIF	leukemia inhibitory factor
LPS	lipopolysaccharide
MCP-1	monocyte chemotactic protein-1
PAF	paraformaldehyde
PBS	Phosphate-buffered saline
PBST	Phosphate-buffered saline with 0.3% Triton X-100
PND	day of postnatal development (postnatal day)
